# Human type I IFN deficiency does not impair B cell response to SARS-CoV-2 mRNA vaccination

**DOI:** 10.1084/jem.20220258

**Published:** 2022-11-07

**Authors:** Aurélien Sokal, Paul Bastard, Pascal Chappert, Giovanna Barba-Spaeth, Slim Fourati, Alexis Vanderberghe, Pauline Lagouge-Roussey, Isabelle Meyts, Adrian Gervais, Magali Bouvier-Alias, Imane Azzaoui, Ignacio Fernández, Andréa de la Selle, Qian Zhang, Lucy Bizien, Isabelle Pellier, Agnès Linglart, Anya Rothenbuhler, Estelle Marcoux, Raphael Anxionnat, Nathalie Cheikh, Juliane Léger, Blanca Amador-Borrero, Fanny Fouyssac, Vanessa Menut, Jean-Christophe Goffard, Caroline Storey, Caroline Demily, Coralie Mallebranche, Jesus Troya, Aurora Pujol, Marie Zins, Pierre Tiberghien, Paul E. Gray, Peter McNaughton, Anna Sullivan, Jane Peake, Romain Levy, Laetitia Languille, Carlos Rodiguez-Gallego, Bertrand Boisson, Sébastien Gallien, Bénédicte Neven, Marc Michel, Bertrand Godeau, Laurent Abel, Felix A. Rey, Jean-Claude Weill, Claude-Agnès Reynaud, Stuart G. Tangye, Jean-Laurent Casanova, Matthieu Mahévas

**Affiliations:** 1 Necker Enfants Malades Institute, INSERM U1151/CNRS UMR 8253, Action thématique incitative sur programme-Avenir Team Auto-Immune and Immune B cell, University Paris Cité, University Paris-Est-Créteil, Créteil, France; 2 Laboratory of Human Genetics of Infectious Diseases, Necker Branch, INSERM U1163, Necker Hospital for Sick Children, Paris, France; 3 Imagine Institute, University Paris Cité, Paris, France; 4 Department of Pediatrics, Necker Hospital for Sick Children, Assistance Publique-Hôpitaux de Paris, Paris, France; 5 St. Giles Laboratory of Human Genetics of Infectious Diseases, Rockefeller Branch, The Rockefeller University, New York, NY; 6 INSERM U955, team 2. Mondor Biomedical Research Institute, Paris-Est Créteil University, Créteil, France; 7 Institut Pasteur, University Paris Cité, CNRS UMR 3569, Structural Virology Unit, Paris, France; 8 Virology, Bacteriology, Hygiene and Mycology-Parasitology, Henri Mondor University Hospital, Assistance Publique-Hôpitaux de Paris, Créteil, France; 9 INSERM U955, team 18. Mondor Biomedical Research Institute, Paris-Est Créteil University, Créteil, France; 10 Departement of Internal Medicine, Henri Mondor University Hospital, Assistance Publique-Hôpitaux de Paris, Paris-Est Créteil University, Créteil, France; 11 Department of Immunology and Microbiology, Laboratory for Inborn Errors of Immunity, Department of Pediatrics, University Hospitals Leuven and Katholieke Universiteit Leuven, Leuven, Belgium; 12 Pediatric Immuno-hemato-oncology Unit, Centre Hospitalier Universitaire Angers, Angers, France; 13 University Angers, Nantes university, Centre Hospitalier Universitaire Angers, INSERM, CRCI2NA, SFR ICAT, Angers, France; 14 Departement of Pediatric Endocrinology, Bicêtre University Hospital, Assistance Publique-Hôpitaux de Paris, Paris Saclay University, Le Kremlin-Bicêtre, France; 15 Department of Pediatrics, Nord Franche Comté Hospital, Trévenans, France; 16 Department of Pediatrics, Besançon Hospital, Besançon, France; 17 Department of Pediatric Endocrinology and INSERM NeuroDiderot, Referral Centre for Endocrine, Growth and Development diseases, Assistance Publique-Hôpitaux de Paris Nord, University of Paris, Paris, France; 18 Department of Internal Medicine, Lariboisière University Hospital, Assistance Publique-Hôpitaux de Paris, University of Paris, Paris, France; 19 Department of Pediatric Hemato-oncology, Childrens Hospital, Nancy University Hospital, Nancy, France; 20 Department of Pediatrics, Mother-Child Hospital, Nantes, France; 21 Department of Internal Medicine, Université Libre de Bruxelles—Hôpitaux Universitaire de Bruxelles, Erasme Hospital, Bruxelles, Belgique; 22 Departement of Pediatric Endocrinology, Robert Debré Hospital, Assistance Publique Hôpitaux de Paris, Paris, France; 23 GénoPsy Referral Center, Centre de Référence de Maladies Rares Rare Disease with Psychiatric Epression, Le Vinatier Hospital, Bron, France; 24 Department of Internal Medicine, Infanta Leonor University Hospital, Madrid, Spain; 25 Neurometabolic Diseases Laboratory, Institut d'Investigació Biomèdica de Bellvitge-Hospital Duran i Reynals, Centro de Investigación Biomédica en Red de Enfermededas Raras U759, and Catalan Institution of Research and Advanced Studies, Barcelona, Spain; 26 University of Paris, University of Paris-Saclay, Université de Versailles Saint-Quentin-en-Yvelines, INSERM UMS11, Villejuif, France; 27 French Blood Agency, La Plaine Saint-Denis, France; 28 UMR1098 RIGHT, INSERM, French Blood Agency, Franche-Comté University, Besançon, France; 29 Department of Immunology and Infectious Diseases, Sydney Children’s Hospital, Randwick, New South Wales, Australia; 30 School of Women’s and Children’s Health, University of New South Wales, Sydney, Australia; 31 Clinical Immunogenomics Research Consortium of Australasia, Sydney, Australia; 32 Queensland Paediatric Immunology and Allergy Service, Children's Health Queensland, Brisbane, Australia; 33 University of Queensland, Brisbane, Australia; 34 Department of Pediatric Immuno-Haematology and Rheumatology, Necker-Enfants Malades University Hospital, Assistance Publique-Hôpitaux de Paris, Paris, France; 35 Department of Immunology, Hospital Universitario de G.C. Dr. Negrín, Canarian Health System, Las Palmas de Gran Canaria, Spain; 36 University Fernando Pessoa Canarias, Las Palmas de Gran Canaria, Spain; 37 Department of Infectious Diseases, Henri Mondor University Hospital, Assistance Publique-Hôpitaux de Paris, Paris-Est Créteil University, Créteil, France; 38 Garvan Institute of Medical Research, Darlinghurst, Sydney, New South Wales, Australia; 39 St Vincent’s Clinical School, Faculty of Medicine & Health, University of New South Wales, Sydney, New South Wales, Australia; 40 Howard Hughes Medical Institute, New York, NY

## Abstract

Inborn and acquired deficits of type I interferon (IFN) immunity predispose to life-threatening COVID-19 pneumonia. We longitudinally profiled the B cell response to mRNA vaccination in SARS-CoV-2 naive patients with inherited TLR7, IRF7, or IFNAR1 deficiency, as well as young patients with autoantibodies neutralizing type I IFNs due to autoimmune polyendocrine syndrome type-1 (APS-1) and older individuals with age-associated autoantibodies to type I IFNs. The receptor-binding domain spike protein (RBD)–specific memory B cell response in all patients was quantitatively and qualitatively similar to healthy donors. Sustained germinal center responses led to accumulation of somatic hypermutations in immunoglobulin heavy chain genes. The amplitude and duration of, and viral neutralization by, RBD-specific IgG serological response were also largely unaffected by TLR7, IRF7, or IFNAR1 deficiencies up to 7 mo after vaccination in all patients. These results suggest that induction of type I IFN is not required for efficient generation of a humoral response against SARS-CoV-2 by mRNA vaccines.

## Teaser

Intact B cell responses to SARS-CoV-2 mRNA vaccines in patients with genetic or acquired type I IFN deficiencies suggest that type I IFNs induced by mRNA vaccines are not required for vaccine efficacy.

## Graphical Abstract

**Figure:**
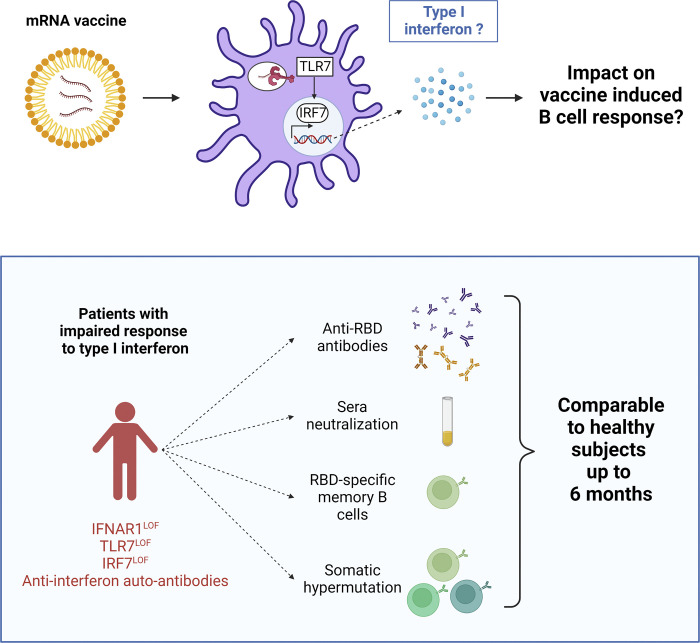

## Introduction

Since their first use in clinical practice in late 2020, SARS-CoV-2 mRNA vaccines have changed the worldwide perspective on the COVID-19 pandemic (Watson et al., 2022). These vaccines have shown excellent clinical efficacy and safety profiles and strong protection against life-threatening severe COVID-19 cases, thus alleviating healthcare systems around the world (Baden et al., 2020; Barda et al., 2021; Polack et al., 2020). The BNT162b2 and mRNA-1273 vaccines contain nucleoside-modified mRNA encoding the original Wuhan-Hu-1 SARS-CoV-2 spike glycoprotein (S) encapsulated in a lipid nanoparticle (Schoenmaker et al., 2021). Both vaccines elicit strong clinical protection along with robust B and T cell responses, including the generation of germinal centers (GCs) producing long-lived plasma cells and memory B cells (MBCs; Cho et al., 2021; Goel et al., 2021; Sokal et al., 2021a; Turner et al., 2021; Kim et al., 2022).

This breakthrough in the fight against SARS-CoV-2 is the result of years of fundamental research (Pardi et al., 2018; Sahin et al., 2014). One key point was the replacement of uridine with 1methyl-pseudo-uridine (m1ψ) to avoid mRNA sensing by the innate immune system’s microbial sensors, stimulation of which by unmodified or unpurified mRNA results in fatal shock in animals (Anderson et al., 2010; Karikó et al., 2005; Karikó et al., 2008; Karikó et al., 2011; Schlee and Hartmann, 2016). Unmodified single-stranded mRNA sensing by TLRs TLR7/TLR8 in endosomes or by retinoic acid–inducible gene I (RIG-1)/melanoma differentiation-associated protein 5 (MDA5) in the cytosol induces type I IFN production through activation of IRF3 and IRF7 (Darnell, 1997; Karikó et al., 2004; Kawasaki and Kawai, 2014; Nelson et al., 2020; Rehwinkel and Gack, 2020; Sahin et al., 2014). In contrast, mRNA vaccines using purified modified mRNA are thought to have limited residual sensing by these sensors (Nelson et al., 2020; Pardi et al., 2018; Stuart, 2021).

Despite the vaccination of billions of individuals, the precise cellular mechanism(s) triggered by mRNA vaccines that lead to such a robust immune response remain(s) unknown. In particular, it is unclear whether any components of mRNA vaccines have an immunostimulatory effect and may act as an intrinsic “adjuvant,” favoring induction of durable immune response (Pardi et al., 2018; Stuart, 2021). Either mRNA molecules themselves or associated lipid nanoparticles may play this role. It has been proposed that modified mRNA may have residual TLR sensing leading to type I IFN production, favoring antigen-presenting cell maturation (Pardi et al., 2018; Sahin et al., 2014; Schoenmaker et al., 2021; Stuart, 2021). To date, this fundamental question has only been recently addressed in a mouse model (Alameh et al., 2021) showing that ionizable lipids from the encapsulated nanoparticle favor the establishment of a GC reaction through IL-6. It also suggests a role for mRNA sensing, especially via TLRs, as MyD88-deficient mice showed diminished B cell responses to vaccines.

Human type I IFNs, which include 13 IFN-α, IFN-β, IFN-ε, IFN-κ, and IFN-ω, play a fundamental role in host defense against some viral infections (Casanova and Abel, 2021; Ciancanelli et al., 2015; Hoffmann et al., 2015; de Weerd et al., 2020; Zhang et al., 2020; Casanova and Abel, 2022). Deficiencies in type I IFN signaling in humans have been shown to underlie life-threatening COVID-19 pneumonia in at least 20% of cases (Zhang et al., 2022). Patients with autoantibodies neutralizing IFN-α, -ω, or -β display increased susceptibility to critical COVID-19 pneumonia (Bastard et al., 2020; Bastard et al., 2021a; Bastard et al., 2021b; Chauvineau-Grenier et al., 2022; Goncalves et al., 2021; Koning et al., 2021; Raadsen et al., 2021; Troya et al., 2021; Vazquez et al., 2021; Wang et al., 2021; van der Wijst et al., 2021; Zhang et al., 2022). Additionally, inborn errors of immunity (IEI) affecting the production of or response to type I IFNs have been identified in previously healthy individuals with life-threatening COVID-19 pneumonia (Asano et al., 2021; Zhang et al., 2020; Zhang et al., 2022). Patients with recessive and complete deficiencies in *TLR7*, *IRF7*, or the type I IFN receptor (heterodimeric IFNAR, encoded by *IFNAR1* and *IFNAR2*) have impaired type I IFN immunity and are at risk of life-threatening viral infections, including COVID-19 pneumonia (Campbell et al., 2022; Hernandez et al., 2019; Zhang et al., 2022). IFNAR1 is essential for cellular responses to the 17 type I IFNs (Bastard et al., 2022a). IRF7 is a transcription factor required for the amplification of human type I and type III (IL29, IL28A, IL28B) IFNs, with the exception of IFN-β. IRF7 is constitutively produced at high levels by plasmacytoid dendritic cells (pDCs), making them the major type I IFN–producing cell type (Casanova and Abel, 2021; Ciancanelli et al., 2015; Österlund et al., 2007). TLR7 is the major endosomal sensor of SARS-CoV-2 in pDCs (Asano et al., 2021; Onodi et al., 2021).

The prevalence of autoantibodies neutralizing IFN-α and/or -ω increases with age in uninfected individuals in the general population, reaching more than 4% of individuals over 70 yr old (Bastard et al., 2021a). They can also be found in early childhood in patients with IEI underlying autoimmunity, including autoimmune polyendocrinopathy syndrome type 1 (APS-1) caused by deleterious germline biallelic *AIRE* mutations (Bastard et al., 2021b; Husebye et al., 2018). These auto-Abs have also been found in about 20% of patients with “breakthrough” COVID-19 hypoxemic pneumonia with seemingly normal antibody response to two injections of RNA vaccine (Bastard et al., 2022b). As these autoantibodies predispose a sizeable proportion of human beings to critical COVID-19, apparently, even in some vaccinated individuals, it is important to determine if they compromise vaccine efficacy. Moreover, testing B cell responses of patients with impaired IFN signaling to mRNA vaccine may inform us as to whether m1ψ mRNA-dependent type I IFNs induction, if any, following its potential in vivo sensing, in particular by pDCs, would have a quantitative or qualitative impact on the immune response to and clinical efficacy by such vaccines. We thus performed an in-depth characterization of the anti–SARS-CoV-2 B cell response to BNT162b2 or mRNA-1273 mRNA vaccines in young and older patients with auto-Abs to type I IFNs due to APS-1 or age-associated autoimmunity, as well as in five patients with recessively inherited, complete IRF7, TLR7, or IFNAR1 deficiency, and compared these responses with young and adult healthy individuals up to 7 mo after mRNA vaccination.

## Results

### mRNA vaccination elicits normal antibody responses in patients with type I IFN deficiency

**Figure 1.**
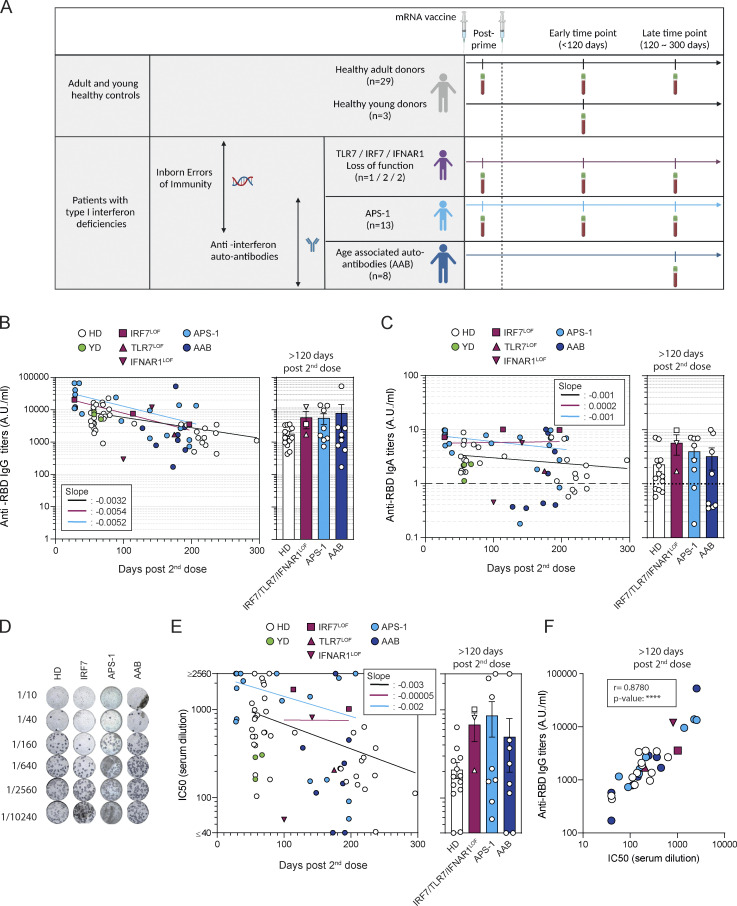
**mRNA vaccines induce robust humoral responses in patients with type I IFN deficiency. (A)** Overview of the study. **(B)** Evolution of anti–SARS-CoV-2 RBD serum IgG titers after mRNA vaccination. IgG titers (arbitrary units, A.U./ml) are shown according to the time of sampling (d from the second dose) for each patient (left panel) and are then compared between groups at late time point (>120 d, right panel): healthy controls (*n* = 33 including HD, white circle, *n* = 29, and YD, green circle), *IRF7*^LOF^ or *TLR7*^LOF^ or *IFNAR1*^LOF^ (purple; *IRF7*^LOF^: square, *n* = 2; *TLR7*^LOF^: up triangle, *n* = 1; *IFNAR1*^LOF^ down triangle, *n* = 2), APS-1 (light blue circle, *n* = 13), and AAB (dark blue circles, *n* = 8). Late time point was performed at mean 221 d, (range 193–296) for HD at 157 and 197 d for the 2 *IRF7*^LOF^ patients, 175 d for the *TLR7*^LOF^ patient, and 142 d for the *IFNAR1*^LOF^ patient, for APS-1 patients at mean 176 d (range 139–207 d) and for AAB mean 180 d, (range 128–211 d). See Table S1 A. Bars indicate mean ± SEM. **(C)** Evolution of anti–SARS-CoV-2 RBD serum IgA titers after mRNA vaccination. IgA titers (A.U./ml) are shown according to the time of sampling (days) for each patient (left panel) and are then compared between groups at late time point (>120 d, right panel) Dotted line indicates positivity threshold. **(D)** Representative wells for the in vitro neutralization assay of sera against D614G SARS-CoV-2 virus for one patient from each cohort. Dark blue spots represent SARS-CoV-2 infected cells. Top-to-bottom wells show increasing serum dilution (dilution is indicated on the left). **(E and F)** Absence of foci indicate virus neutralization. **(E)** Evolution of IC_50_ against D614G SARS-CoV-2 after mRNA vaccination of sera tested from HD (*n* = 31), *IRF7*^*LOF*^*/TlR7*^*LOF*^*/IFNAR1*^*LOF*^ (*n* = 3), APS-1 (*n* = 13), and AAB (*n* = 8). IC_50_ (1/dilution) are shown according to the time of sampling (days) for each patient (left panel) and are then compared between groups at late time point (>120 d, right panel). **(F)** Correlation between the anti–SARS-CoV-2 RBD IgG titers at late time point and neutralization potency of the sera at late time point. We performed nonlinear regression in B, C, and E (left panels; semilog line, x is linear, y is log), slopes of the lines are indicated in the boxes; and Kruskal–Wallis with multiple comparisons with Dunn’s Correction in B, C, and E (right panels). All were nonsignificant (P > 0.05). In F, Spearman correlation was performed. P value <0.0001.

**Figure S1.**
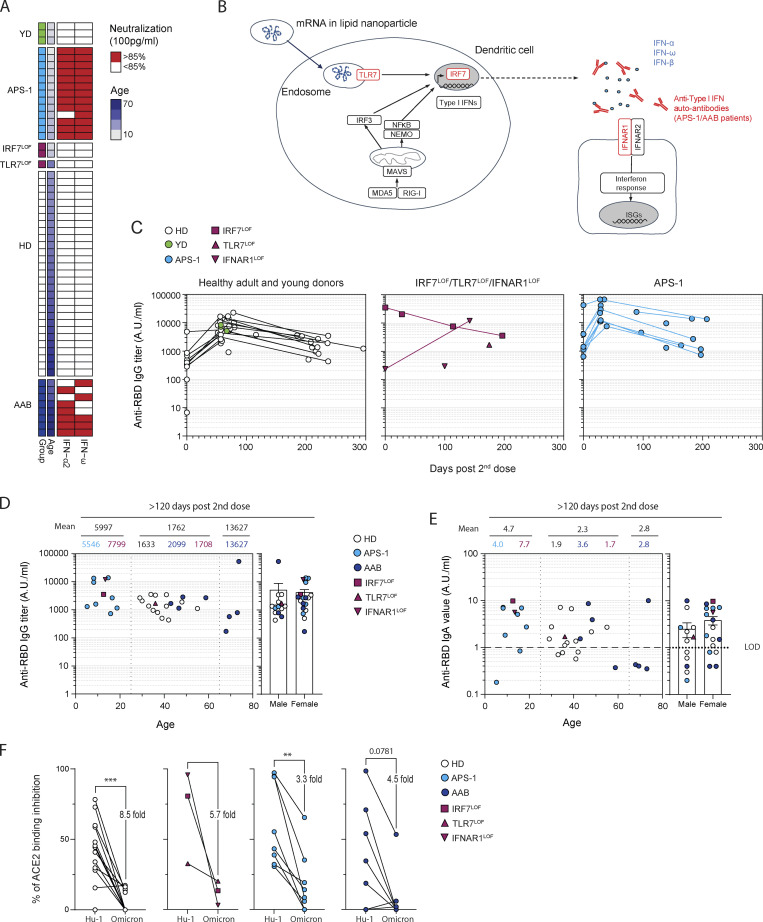
**mRNA vaccines induce robust humoral response in patients with type I IFN deficiency. (A)** Heatmap showing the neutralization potency of the patients’ sera against IFN-α-2 and -ω at 100 pg/ml. Each line represents one patient. First column indicates patient group (healthy controls are in green [YD] or white [HD], APS-1 patients are in light blue, patient with *IRF7*^LOF^ or *TLR7*^LOF^ in purple, and patient with AAB in dark blue), second line indicates age, and the two last columns indicate sera neutralization. Sera above the positivity threshold (>85%) are depicted in red. Sera below the positivity threshold are depicted in white. **(B)** Schematic representation of type I IFN response theoretically triggered by mRNA vaccines. Steps that are impaired in one of the studied cohorts are indicated in red. NEMO, NF-κB essential modulator; MAVS, mitochondrial antiviral signaling protein; ISG, IFN stimulated genes. **(C)** Longitudinal evolution of anti–SARS-CoV-2 RBD serum IgG titers (A.U./ml) after mRNA vaccination in patients from each cohort according to the day of sampling. Day 0 represents IgG titers after the prime and before the second dose. **(D)** Anti RBD IgG titers (A.U./ml) at late time point (>120 d) according to the age (left panel) or sex (right panel) of the patient. Numbers on the top of the left panel indicate the mean for age group (0–25; 25–65; >65 yr old) and above number indicate the mean for each cohort in each age group. **(E)** Anti RBD IgA titers (A.U./ml) at late time point (>120 d) according to the age (left panel) or sex (right panel) of the patient. Numbers on the top of the left panel indicate the mean for age group (0–25; 25–65; >65 yr-old) and above number indicate the mean for each cohort in each age group. LOD, limit of detection. **(F)** Percentage of ACE2 binding inhibition using competitive ELISA with the sera from the patient at late time point against the ancestral Hu-1 RBD or the Omicron BA.1 RBD. Mean decrease in percentage of inhibition is indicated on the figure. We performed Mann–Whitney in D and E, and Wilcoxon test in F (***P < 0.001, **P < 0.01).

To study the response to mRNA vaccines in SARS-CoV-2 naive individuals with type I IFN deficiencies, we first assessed a cohort of 13 patients with APS-1 (median age: 9, range 2–18 yr) and detectable neutralizing auto-Abs against IFN-α and -ω, and another cohort of eight patients with age-associated autoantibodies (AABs) to IFN-α and/or ω (median age: 63, range 43–76 yr; 3/8 neutralized IFN-α only, 2/8 IFN-ω only, and 3/8 both at 100 pg/ml; Fig. 1 A and Fig. S1, A and B; and see Table S1 A). We also enrolled five patients with very rare IEI linked to type I IFN induction or signaling (Fig. 1 A and Fig. S1 B; and see Table S1 A); a 5- and 12-yr-old female patient homozygous for a loss-of-function (LOF) mutation in *IRF7,* a 36-yr-old male patient hemizygous for a LOF *TLR7* mutation, and a 4-yr-old male and a 13-yr-old female patient homozygous for a LOF *IFNAR1* mutation (nonsense mutation p.Glu386*; Asano et al., 2021; Bastard et al., 2022a; Campbell et al., 2022; Ciancanelli et al., 2015). The humoral response elicited in these patients was compared with that in three young (aged 6–10 yr) and 29 adult healthy individuals (median age: 41, range 26–60 yr; Fig. 1 A and see Table S1 A), including 23 individuals previously sampled 2 mo after mRNA vaccination (Sokal et al., 2021a). None of these young and adult healthy donors (respectively YDs and HDs) had detectable neutralizing auto-Abs against IFN-α or -ω (Fig. S1 A). All patients were naive for previous SARS-CoV-2 infection and received two doses of the BNT162b2 (57/58) or the mRNA-1273 (1/58, see Table S1 A) vaccines, 4 wk apart. The first injection is referred to as the priming dose. Patients were sampled at several time points from priming up to 7 mo after the second dose (Fig. 1 A and see Table S1 A). Healthy adult individuals in our main cohort were sampled, when possible, 62 (±SD: 8.9) and 214 (±SD: 37.2) days after the second vaccine dose. Sampling of patients with inborn type I IFN deficiencies was performed alongside routine appointments to limit as many as possible hospital visits and was, therefore, more heterogeneous. Individuals with acquired anti-IFNs antibodies were sampled at a late time point (>120 d) following the second vaccine dose.

All patients in these cohorts displayed a robust early RBD-specific IgG response following the second vaccine dose (Fig. 1 B, Fig. S1 C, and Table S1 B), except one of the two *IFNAR1*^LOF^ patients whose response was lower than expected at the time of sampling (day 100 after the second dose). At late time points (>120 d after the second dose), no difference in anti-RBD IgG titers was observed between groups, and all patients displayed detectable titers (Fig. 1 B and Fig. S1 C). These titers did not correlate with age or sex (Fig. S1 D). mRNA vaccine also elicited RBD-specific IgA responses in all patients, but with lower overall titers, which fell below detectable levels in 4/14 HD, 2/9 APS-1, and 4/8 AAB patients at the last time point in our study (Fig. 1 C). As for observed IgG titers, the overall heterogeneity seen at these late time points for IgA titers could not be explained simply by age- or sex-related differences, although we did observe increased heterogeneity in patients >65 yr old (Fig. S1 E). Finally, sera from most sampled patients displayed in vitro neutralization potential against the authentic D614G SARS-CoV-2, with half maximal inhibitory concentration IC_50_ > 1/40; this was observed for up to 7 mo after the second dose in most patients (Fig. 1, D and E). In line with the overall reduction in serum IgG titers, IC_50_ decreased significantly with time in all groups, with again some level of heterogeneity in each group at late time points but overall strong correlation between IC_50_ and anti-RBD IgG titers in all groups (Fig. 1 F), confirming RBD as the major target of the neutralizing antibody response in all studied individuals. The potency of sera against the BA.1 (Omicron) SARS-CoV-2 variant was further estimated using binding competitive ELISA against Omicron or Hu-1 RBD. At late time points, Omicron RBD binding inhibition appeared similarly reduced in all patients and cohorts (Fig. S1 F). Overall, these results suggest that both rapid extrafollicular responses and long-lasting IgG production can be induced in patients with type I IFN deficiencies and appear to follow similar kinetics to those described in the healthy population. The reduced IgG titers seen in the prime or early sampling time points of the *IFNAR1*^LOF^ patients could be related to dampened extrafollicular responses in these patients in the absence of any IFN-β signaling, as previously hypothesized (Goldberg et al., 2021; Levin et al., 2021; Pegu et al., 2021; Thomas et al., 2021). However, it should be noted that one of these two patients nonetheless displayed strong IgG titers, IC_50_ and Hu-1 RBD binding inhibition potential when sampled later in the response (day 142; Fig. S1, C and F). The second patient was unfortunately not available for future samplings.

### mRNA vaccinated type I IFN–deficient patients generate normal MBC responses with evidence of prolonged germinal center maturation

**Figure 2.**
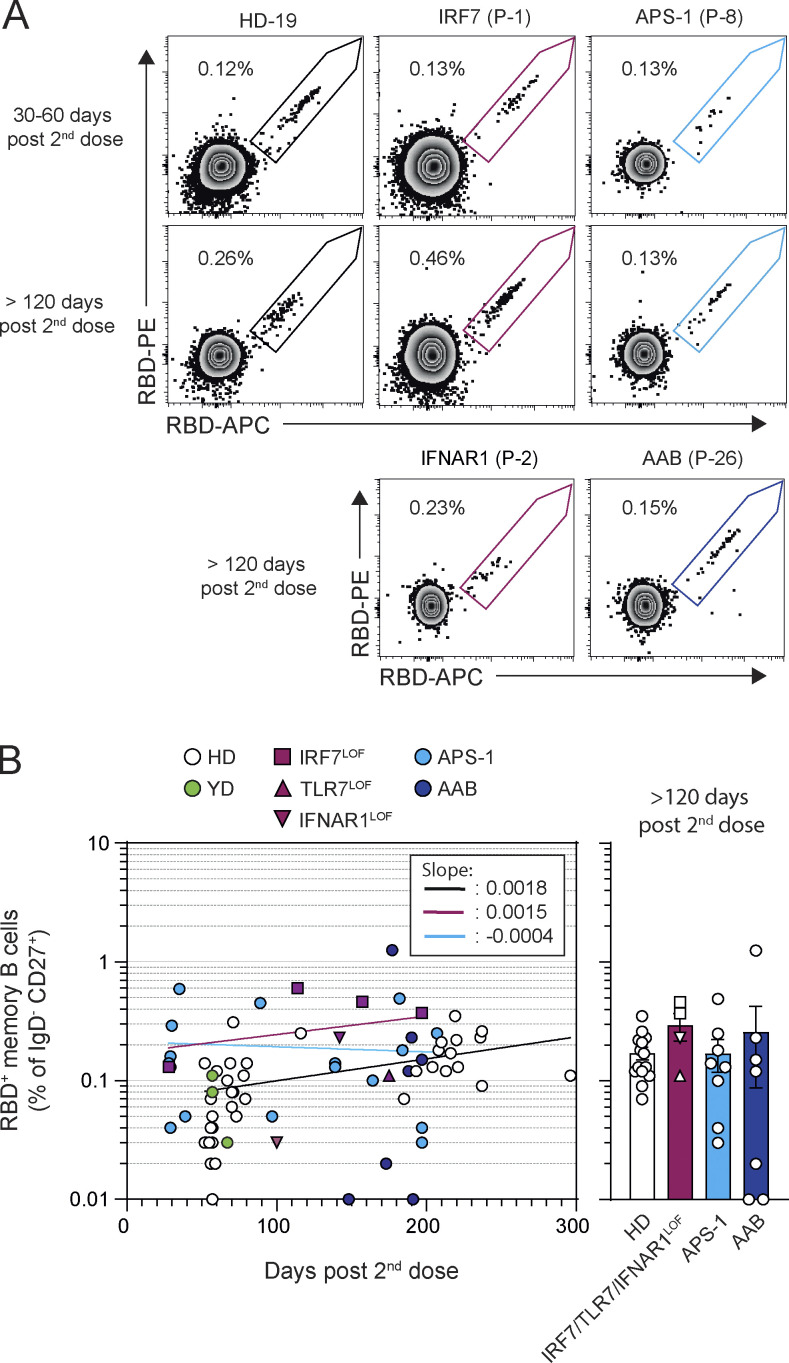
**Circulating MBCs are detected up to 6 mo after mRNA vaccination of patients with type I IFN deficiency. (A)** Representative dot plot of SARS-CoV-2 RBD-staining of CD19^+^IgD^−^CD27^+^CD38^int/^^−^ MBCs 30–60 d and >120 d after the second dose in patients representative of each cohort. **(B)** Evolution of percentage of RBD-specific MBCs among CD27^+^IgD^−^MBCs after mRNA vaccination. Frequencies of RBD-specific MBCs are shown according to the time of sampling (days) for each patient (left panel) and are then compared between groups at late time point (>120 d, right panel). Bars indicate mean ± SEM. We performed nonlinear regression in B (semilog line, x is linear, y is log); slopes of the lines are indicated in the box (left panel). Kruskal–Wallis with multiple comparisons with Dunn’s Correction, all nonsignificant (P > 0.05, right panel).

**Figure S2.**
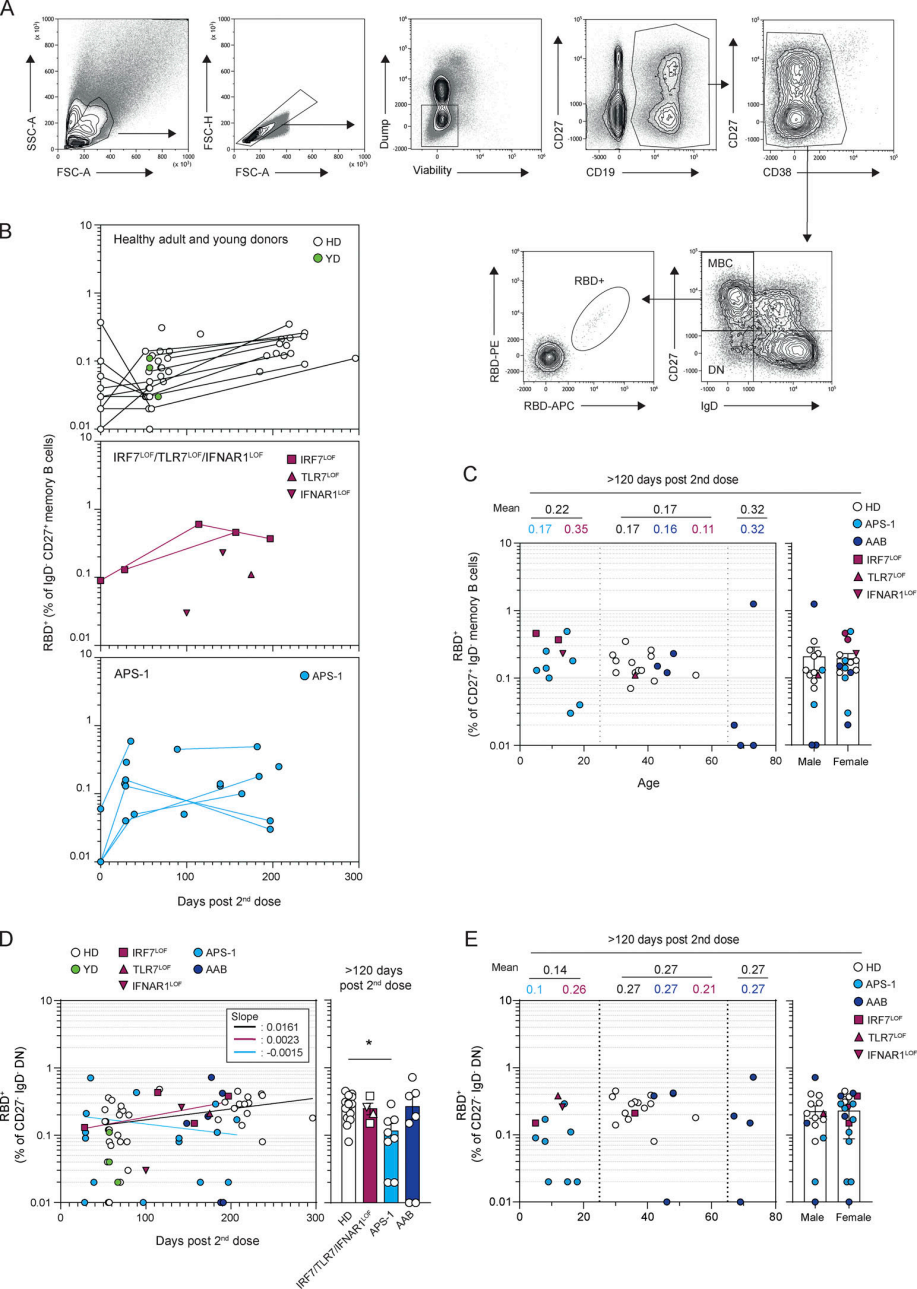
**Circulating MBCs are detected up to 6 mo after mRNA vaccination of patients with type I IFN deficiency. (A)** Flow cytometric gating strategy for the analysis and sorting of SARS-CoV-2 S or RBD-specific MBCs from PBMCs. Lymphocytes were first gated based on morphology, before exclusion of doublets, dead cells, and CD3/CD14 cells. Total CD19^+^ cells were then gated and subdivided into CD38^int/−^ cells and CD27^+^CD38^hi^ plasma cells. CD38^int/−^ B cells were further divided in four quadrants using CD27 and IgD staining. Upper left quadrant defines MBCs, lower left quadrant DN, upper right quadrant CD27^+^IgD^+^ cells, and lower right quadrant naive B cells. SARS-Cov-2 RBD-specific B cells were then analyzed within the B cell population of interest using a His-tagged SARS-Cov-2 RBD protein further revealed by two fluorescently labeled anti-His antibodies. **(B)** Longitudinal evolution of the RBD-specific CD27^+^IgD^−^ MBC (% of CD27^+^IgD^−^ MBCs) in patients from in each cohort. **(C)** Percentage of RBD-specific MBCs at late time point (>120 d) according to the age (left panel) or sex (right panel) of the patient. Numbers on the top of the left panel indicate the mean for age group (0–25; 25–65; >65 yr old) and above numbers indicate the mean for each cohort in each age group. **(D)** Evolution of percentage of RBD-specific DN among CD27^−^IgD^−^DNs after mRNA vaccination. Frequencies of RBD-specific DNs are shown according to the time of sampling (days) for each patient (left panel) and are then compared between groups at late time point (>120 d, right panel). Bars indicate mean ± SEM. **(E)** Percentage of RBD-specific DNs at late time point (>120 d) according to the age (left panel) or sex (right panel) of the patient. Numbers on the top of the left panel indicate the mean for age group (0–25; 25–65; >65 yr old) and above numbers indicate the mean for each cohort in each age group. We performed Mann–Whitney in B, C, and E and nonlinear regression in D (semilog line, x is linear, y is log); slopes of the lines are indicated in the box (left panels). Kruskal–Wallis with multiple comparisons with Dunn’s Correction (right panel). *P < 0.05.

RBD-specific MBCs represent a large fraction of the neutralizing MBC pool against SARS-CoV-2 (Sokal et al., 2021b; Sokal et al., 2021a; Tong et al., 2021). In all HDs, RBD-specific MBCs increased up to 6 mo and showed a twofold increase between 2 and 6 mo after the second dose (Fig. 2, A and B; Fig. S2, A and B; and Table S1 B), consistent with previous reports suggesting a persistent output from ongoing GCs (Cho et al., 2021; Goel et al., 2021; Kim et al., 2022). RBD-specific MBCs were robustly induced by the second dose of the mRNA vaccine in *IRF7*^LOF^, *TLR7*^LOF^, or *IFNAR1*^LOF^ patients and in patients with neutralizing auto-Abs against type I IFNs, whether due to APS-1 or age-dependent autoimmunity, apart from two patients around 70 yr-old with AAB and RBD-specific MBCs below ≤0.01% of the IgD^−^CD27^+^ subset (Fig. 2, A and B; and Fig. S2, B and C). RBD-specific MBCs frequencies remained stable over time in these patients and reached similar levels to those found in HD controls at late time points (Fig. 2, A and B; and Fig. S2, B and C). Comparable frequencies of RBD-specific cells in the double negative (DN) IgD^−^CD27^−^ subset were also detected in all groups (Fig. S2 D), except in APS-1 in whom they appeared to be reduced at a late time point (> 120 d), albeit with strong heterogeneity between patients that did not correlate with age or sex.

**Figure 3.**
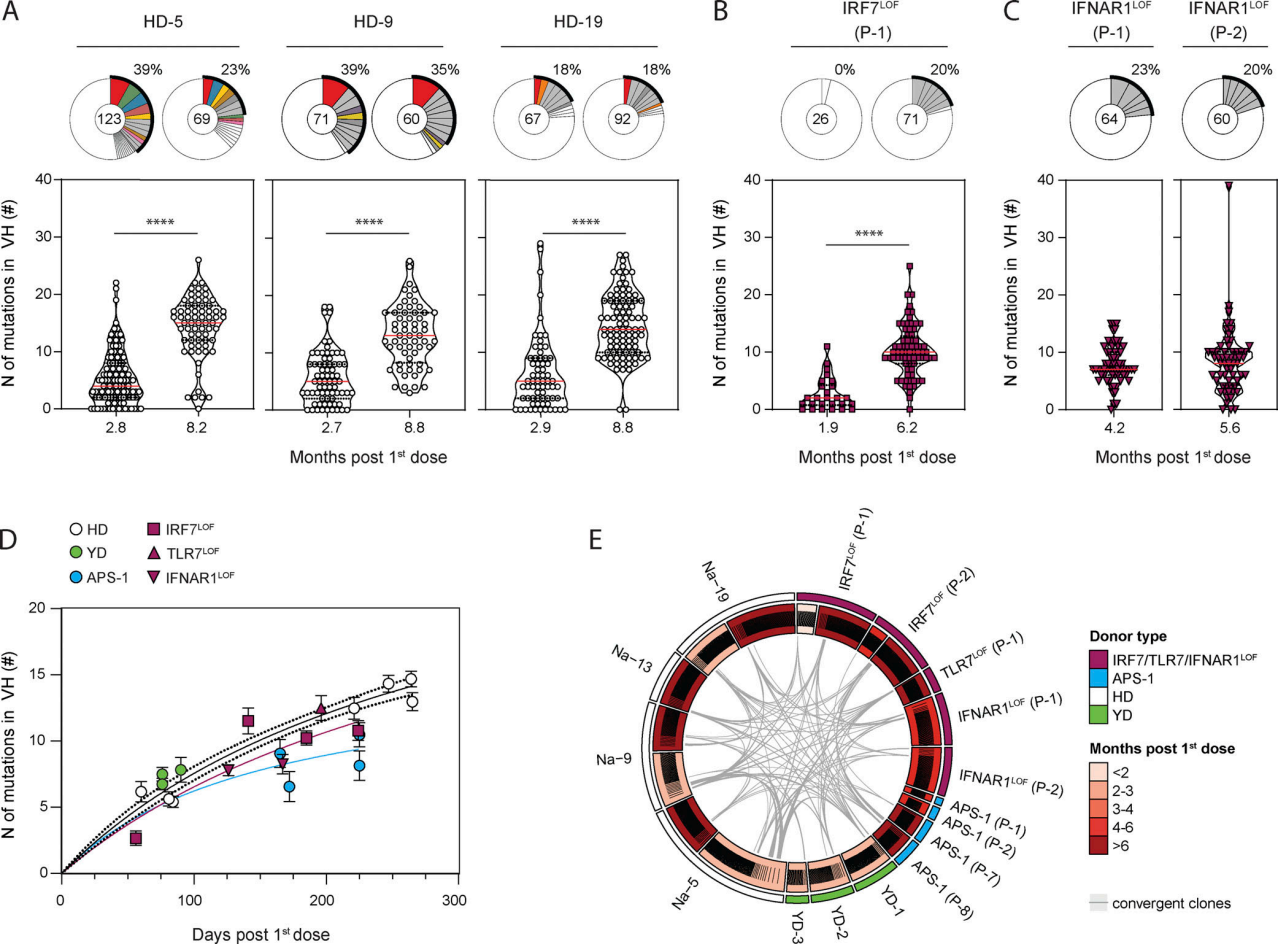
**RBD-specific MBCs are GC derived in patients with type I IFN deficiency. (A–C)** Violin plot showing the number of mutations in the IgV_H_ of RBD-specific MBCs from (A) HD, (B) *IRF7*^LOF^, and (C) *IFNAR1*^LOF^ subjects. All subjects received two doses of mRNA vaccine. Time point of analysis is however indicated here from the first dose (months), as it represents the first antigen encounter. Each box is a single patient. The pie chart at the top of each plot represents the clonal distribution of RBD-specific MBCs at indicated time points. Colored slices indicate an expanded MBC clone found at several time points from the same donor, gray slices indicate an expanded clone not found at another time point from the same donor, and white slices indicate unique sequences but found at another time point from the same donor. Outer black semicircular line indicates the proportion of sequences belonging to expanded clones. The total number of sequences is indicated at the pie center. **(D)** Mean (± SEM) number of mutations in IgV_H_ of RBD-specific MBCs for each analyzed patient at a given time point, plotted according to the time of sampling after the first dose. **(E)** Circos plot showing clonal relationships between RBD-specific MBCs from HD (white outer line for HD, green outer line for YD) and patient with impaired IFN signaling (purple for *IRF7*^LOF^, *TLR7*^LOF^, and *IFNAR*1^LOF^, light blue for APS-1) at indicated time point. Inner line indicates clone distribution for each patient and is colored according to the time of sampling. Gray lines indicate public clones shared between patients. We performed Mann–Whitney in A–C (****P < 0.0001) and nonlinear regression with Michaelis–Menten model in D.

**Figure S3.**
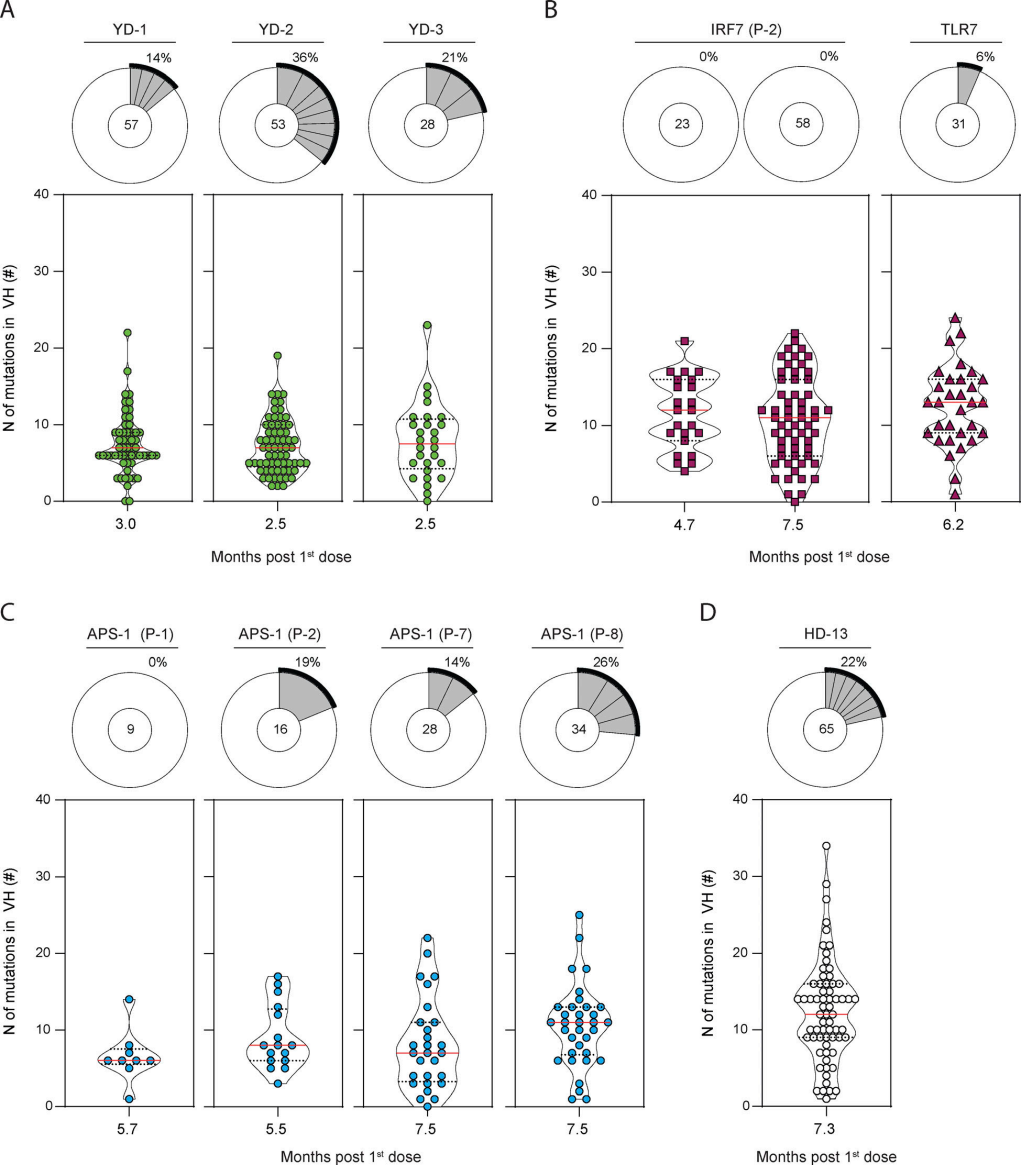
**RBD-specific MBCs are GC derived in patients with type I IFN deficiency. (A–D)** Violin plot showing the number of mutations in the IgV_H_ of RBD-specific MBCs at indicated time point after first dose (months) for YDs (A), IRF7^LOF^ (B), APS-1 (C), and HD (D). Pie chart at the top of each plot represents the clonal distribution of RBD-specific MBCs at indicated time point. Gray slices indicate expanded clones. Outer black semi-circular line indicates the proportion of sequences belonging to expanded clones. The total number of sequences is indicated at the pie center. We performed Mann–Whitney in B (left).

We and others have previously shown that the primary B cell response against RBD largely consisted of unmutated naive B cells in all donors, and its limited homology to previous human coronavirus RBD makes it a neoantigen for the immune system (Gaebler et al., 2021; Hicks et al., 2021; Robbiani et al., 2020; Sokal et al., 2021b; Sokal et al., 2021a). Longitudinal analysis of the heavy chain of the immunoglobulin (IgV_H_) sequences of RBD-specific MBCs from three HDs confirmed the continuous acquisition of somatic mutations for more than 3 mo and up to 8 mo after the initial priming event (i.e., the first dose of mRNA vaccine; Fig. 3 A and Table S1 C), as previously described by others (Cho et al., 2021; Goel et al., 2021; Turner et al., 2021; Kim et al., 2022). A similar increase in mutational load could be detected in one of the *IRF7*^LOF^ patients from which we could analyze RBD-specific MBCs both at an early (1.9 mo after the first dose) and a late (6.2 mo after the first dose) time point (Fig. 3 B). Most importantly, all *IRF7*^LOF^, *TLR7*^LOF^, or *IFNAR1*^LOF^ patients analyzed harbored RBD-specific MBCs with the average number of IgV_H_ mutations matching those detected in RBD-specific MBCs from YDs and HDs at comparable times of sampling after the first vaccine dose (Fig. 3, C and D; Fig. S3, A–D; and Table S1 C). Clear acquisition of IgV_H_ mutations in RBD-specific MBCs could also be detected in APS-1 patients, although, based on our data, one cannot exclude a slightly blunted somatic hypermutation process in these patients (Fig. 3 D and Fig. S3 C). Nonetheless, these data still demonstrate that a lasting germinal center response can be induced in all type I IFN–deficient patients after mRNA vaccination, with clones acquiring high mutational load in all groups.

As previously reported for SARS-CoV-2–infected patients, the overall diversity of the MBC repertoire is high and stable after mRNA vaccination in HDs, with no major clonal expansion detected (Cho et al., 2021). Persisting clones were found in all individuals between 2–3 and 8 mo after the first dose (Fig. 3 A). Despite a limited number of sequences, repertoire analysis of patients with *IRF7*^LOF^, *TLR7*^LOF^, *IFNAR1*^LOF^, or APS-1 also suggested the generation of a polyclonal RBD-specific MBC response (Fig. 3, B and C; and Fig. S3, A–D). Furthermore, convergent RBD clones shared between *IRF7*^LOF^, *TLR7*^LOF^, *IFNAR1*^LOF^, APS-1, and HDs (Fig. 3 E) could be detected in all patients, in line with several studies showing that both natural infection with SARS-CoV-2 and SARS-CoV-2–specific mRNA vaccines elicited a highly convergent response in individuals (Cho et al., 2021; Robbiani et al., 2020).

Altogether, these results show that mRNA vaccines induce a robust GC-derived B cell response in patients with type I IFN deficiencies, which is quantitatively and qualitatively similar to that seen in healthy individuals.

## Discussion

mRNA vaccines against SARS-CoV-2 have reached a milestone in the current COVID-19 pandemic and are now being developed for various infectious and noninfectious diseases (Krienke et al., 2021; Sajid et al., 2021). Our study provides a reassuring picture regarding the vaccine-induced immune response and protection in patients with inherited TLR7, IRF7, or IFNAR1 deficiency. By extension, our data also provide evidence of the efficacy of mRNA vaccines in patients with other inborn errors of type I IFN immunity and in patients with APS-1, whose early-onset neutralizing autoantibodies against IFN-α and -ω are responsible for life-threatening COVID-19 (Bastard et al., 2021b; Beccuti et al., 2020; Hetemäki et al., 2021; Meisel et al., 2021; Zhang et al., 2022). Long-term humoral and MBC responses in these patients were in general quantitatively similar to HDs. It can be noted that MBC responses in young children (i.e., <10 yr), both in patients and in healthy controls, also appeared very similar to those observed in adults, consistent with previous serological reports (Ali et al., 2021; Walter et al., 2022). Most importantly, all patients appeared to acquire MBCs harboring somatic mutations, hallmark of a GC response, suggesting qualitatively similar MBC responses. Data from patients for whom we were able to perform longitudinal sampling suggest sustained GC responses over several months after the second vaccine dose, as previously described after SARS-CoV-2 infection (Cho et al., 2021; Gaebler et al., 2021; Sokal et al., 2021a, 2021b; Turner et al., 2021; Kim et al., 2022). Overall, the only group in which the anti–SARS-CoV-2 MBC response appeared more heterogenous is the elderly population group with autoantibodies neutralizing type I IFNs. Since MBCs responses in both young individuals with type I IFN deficiency and adults <65 yr old with autoantibodies neutralizing type I IFNs were normal, we believe that this most likely points toward an effect of aging on the immune system, independent of a weak type I IFN immunity.

Underlying immune mechanisms triggered by mRNA vaccines have so far only been partially elucidated. Modified mRNAs have been designed to reduce stimulation by microbial sensors, and initial in vitro experiments on DCs described neither type I IFN secretion nor transcription of type I IFN–associated genes (Karikó et al., 2011). Paradoxically, recent work showed that *MyD88*-deficient mice with impaired endosomal sensing of RNA, but not mitochondrial antiviral-signaling–deficient mice with impaired RIG-I and MDA5 cytosolic sensing of RNA, displayed diminished responses to mRNA vaccines (Alameh et al., 2021). As MyD88 is downstream and crucial for endosomal TLR7, 8, and 9 signaling, it suggested that either m1ψ mRNA is still partially recognized by these TLRs or that alternative sensing of modified mRNA or its degradation products can lead in fine to the activation of the IL-1R family downstream signaling pathways (Tahtinen et al., 2022). Li et al. found that mRNA sensing through MDA5 was important for CD8^+^ T cell responses to the BNT162b2 mRNA vaccine, although MDA5 is mainly predicted to recognize long double-stranded RNA (Li et al., 2022). Of note, many cytokines are also induced after mRNA vaccination, both at the systemic and local level (Bergamaschi et al., 2021; Tahtinen et al., 2022). Their beneficial or detrimental role in supporting the establishment of a protective adaptive anti–SARS-CoV-2 immune response, however, still needs to be deciphered.

Type I IFN has been reported as important for B and T cell responses against adenoviruses in mice as well as in the formation of tertiary lymphoid structures and antibody responses (Denton et al., 2019; Zhu et al., 2007). Our study suggests that baseline or mRNA-vaccine-induced type I IFNs is not essential to achieve optimal maturation of the anti–SARS-CoV-2 B cell response after mRNA vaccination in humans, as best seen in *TRL7*^LOF^, *IRF7*^LOF^, or *IFNAR1*^LOF^ patients. Similar results were obtained in APS-1 patients, but with possibly slightly reduced overall mutational loads. These patients harbor autoantibodies against various other cytokines involved in the immune response (including, but not limited to, IL6, IL-22, or IL17A and F). Thus, it is impossible to precisely pinpoint the cause for such observation and could be resolved by studying patients with IEI that predominantly affect these pathways (Husebye et al., 2018; Meyer et al., 2016). The weak humoral response observed at early time points after mRNA vaccination in *IFNAR1*^LOF^ patients, which contrasts with the initial normal response in APS-1 and *IRF7*^LOF^ patients, could also reflect a role of IFN-β in driving the extrafollicular response, as previously hypothesized (Goldberg et al., 2021; Levin et al., 2021; Pegu et al., 2021; Thomas et al., 2021).

Our work, nonetheless, includes several limitations, mostly linked to the rarity of samples available from patients with type I IFN deficiencies and the fact that these patients were mostly preserved from hospital visits to avoid SARS-CoV-2 infection. While we tried to match and report as much as possible potential variability introduced by differences in time of sampling or donor-related parameters (age, sex), the heterogeneity for these parameters clearly increased in our type I IFN–deficient patient cohorts as compared to our healthy control cohort. Comparison of the humoral response induced by mRNA vaccine and natural infection in type I IFN–deficient patients would also have been interesting to decipher the role of innate immune sensing in the context of the whole virus. However, we were limited in the ability to retrospectively collect samples from severe patients with type I IFN autoantibodies infected during the first wave of the pandemic. This, plus the limited numbers of patients in certain patient groups, may have prevented us from highlighting small differences in the generated humoral response. We, however, feel strongly that the global picture shown by this study is one of a functional vaccine-induced humoral immune response in all groups. Finally, one should underline that depending on various parameters, such as affinity and neutralization potency of the auto-Abs against type I IFNs, protection conferred by vaccination may be reduced in AAB patients (Bastard et al., 2022b).

Altogether, our results suggest that GC-derived maturation processes are intact in patients with genetic or acquired type I IFN deficiency. Since high-affinity matured MBCs likely produce potent neutralizing antibodies against SARS-CoV-2 variants of concern, this suggests that a third dose of mRNA vaccine would be particularly beneficial in this population prone to develop severe forms of COVID-19 (Cho et al., 2021; Sokal et al., 2021a; Sokal et al., 2022). This is particularly relevant as patients with a seemingly normal antibody response to two doses of RNA vaccine can develop “breakthrough” COVID-19 hypoxemic pneumonia. We even found that about 20% of such patients in one cohort were critically ill because of high levels of auto-Abs neutralizing high concentrations of type I IFNs (Bastard et al., 2022b). These findings thus have major fundamental and clinical implications for mRNA vaccines as well as the management of patients with inherited or acquired deficiencies in type I IFN immunity.

## Materials and methods

### Study participants

All “naive” patients from the MEMO-COV-2 cohort sampled at least once ∼2 mo after the second dose were included in this study as HDs and sampled anew 221 d (mean, ± SD: 26 d) after the second dose with six additional SARS-CoV-2 naive patients. These subjects were mainly healthcare workers who had no history of COVID-19 and negative IgG antinucleocapsid (and/or spike). We further included three young donors aged 6, 6, and 10 yr old and sampled, respectively, 57, 57, and 67 d after the second dose. Altogether, these 32 patients constituted the adults and young HDs cohort (IRB 2018-A01610-55). Detailed information on the individuals, including gender and health status, can be found in Table S1.

All patients from this cohort received two doses of the BNT162b2 mRNA vaccine 26.7 ± 3.1 d apart. All patients vaccinated with the BNT162b2 received the 30-µg dosage of mRNA, except the three young donors and the 4-yr-old INFAR1^LOF^ patient (P-1) who received the 10-µg dosage. The patient who received the mRNA-1273 vaccine received the 50-µg dosage. Clinical and biological characteristics of these patients are summarized in Table S1 A. Patients were recruited at the Henri Mondor University Hospital (Assistance Publique-Hôpitaux de Paris) between January and November 2021. MEMO-COV-2 study (NCT04402892) was approved by the ethical committee Ile-de-France VI (Number: 40-20 HPS) and was performed in accordance with the French law. Written informed consent was obtained from all participants.

Patients from the *IRF7*^LOF^/*TlR7*^LOF^/*IFNAR1*^LOF^, APS-1, and AAB cohorts were recruited through the practitioner taking care of them during their routine followups and sampled at one or several time points (see Table S1 A). These subjects had no history of COVID-19 and negative IgG anti-nucleocapsid (and/or spike) and received two doses of the BNT162b2, except one who received two doses of the mRNA-1273 mRNA vaccine.

Late time sampling (>120 d after the second dose), which was used for the comparative analysis, was performed at mean 221 d (range 193–296) for HD, at 157 and 197 d for *IRF7*^LOF^ patients, 175 d for *TLR7*^LOF^ patient, 142 d for *IFNAR1*^LOF^ patient, 176 d (range 139–207 d) for APS-1 patients, and 180 d (range 128–211 d) for AAB (Table S1 A)

For all patients, the “prime” time point was defined as the sampling between the two doses.

Written informed consent was obtained from patients or their parents in the country in which they were followed, in accordance with local regulations. The study was approved by the institutional review boards of The Rockefeller University and Institut National de la Santé et de la Recherche Médicale (INSERM). Experiments were conducted in the United States and France in accordance with local regulations and with the approval of the institutional review boards of The Rockefeller University and INSERM.

### Virus strains

The reference D614G strain (hCoV-19/France/GE1973/2020) was supplied by the National Reference Centre for Respiratory Viruses hosted by Institut Pasteur and headed by Sylvie van der Werf. The viral strains were supplied through the European Virus Archive goes Global platform, a project that has received funding from the European Union’s Horizon 2020 research and innovation program under grant agreement number 653316. D614G viral stocks at passage 2 were prepared by amplification and titration in Vero E6 cells. Single-use aliquots stored at −80°C were used for all the assays.

### Anti-RBD (S) SARS-CoV-2 antibodies assay

Serum samples were analyzed for anti-S-RBD IgG titration with the SARS-CoV-2 IgG Quant II assay (ARCHITECT, Abbott Laboratories). The latter assay is an automated chemiluminescence microparticle immunoassay that quantifies anti-RBD IgG with 50 AU/ml as the positive cutoff and a maximal threshold of quantification of 40,000 AU/ml. Dilutions were performed if necessary. All assays were performed by trained laboratory technicians according to the manufacturer’s standard procedures.

Additionally, anti–SARS-CoV-2 IgA was measured using ELISA targeting spike protein subunit 1 (Euroimmun Medizinische Labordiagnostika) according to the manufacturer’s instructions. Briefly, diluted serum samples are added to wells coated with recombinant SARS-CoV-2 antigen and incubated for 60 min at 37°C. After washing, HRP-conjugated anti-human IgA is added for subsequent incubation for 30 min at 37°C. Wells are then washed three times and a chromogen solution is added to each well to stop the reaction. The resultant absorbance is determined on a microplate reader at 450 nm.

### SARS-CoV-2 and BA.1 SARS-CoV-2 binding inhibition

Sera neutralization potency against Hu-1 and BA.1 SARS-CoV-2, presented in Fig. S1 F, was performed using the SARS-CoV-2 Wild Type and SARS-CoV-2 B.1.1.529 Neutralizing Antibody Titer Serologic Assay Kit from Acrobiosystems according to the manufacturer’s instruction (RAS-N022 and RAS-N056). For this competitive ELISA, sera from patients were diluted at 1/10 and incubated with HRP conjugated Hu-1 or BA.1 RBD on a plate precoated with hACE2. After ELISA revelation, the percentage of inhibition was defined as (OD of sample−OD of negative control)/OD negative control.

### Recombinant protein purification

#### Construct design

The SARS-CoV-2 RBD was cloned in pcDNA3.1(+) encompassing the spike (S) residues 331–528, and it was flanked by an N-terminal IgK signal peptide and a C-terminal thrombin cleavage site followed by Hisx8, Strep, and Avi tags.

#### Protein expression and purification

The plasmids coding for recombinant proteins were transiently transfected in Expi293F cells (Thermo Fisher Scientific) using FectoPRO DNA transfection reagent (Polyplus) according to the manufacturer’s instructions. The cells were incubated at 37°C for 5 d and then the culture was centrifuged and the supernatant was concentrated. The proteins were purified from the supernatant by affinity chromatography using His-Trap Excel columns (Cytiva). A final step of size-exclusion chromatography in PBS was also performed using a Superdex200 10/300 (Cytiva).

### Flow cytometry and cell sorting

Peripheral blood mononuclear cells (PBMCs) were isolated from venous blood samples via standard density gradient centrifugation and used after cryopreservation at −150°C. Cells were thawed in RPMI-1640 (Gibco)-10% FBS (Gibco), washed twice, and incubated with 5 µg of the SARS-CoV-2 His-tagged spike protein or WT RBD in 100 µl of PBS (Gibco)-2% FBS during 20 min on ice. For each condition, 2.5 × 10^6^ cells were washed and resuspended in the same conditions and then the fluorochrome-conjugated antibody cocktail including the two anti-His antibodies was added at pretitrated concentrations (1:100 for CD19, CD21, CD11c, CD71, CD38, CD3, CD14, and IgD, 1:50 for CD27, and 1:33 for anti-His tag; Table S1 E) for 20 min at 4°C, and viable cells were identified using a LIVE/DEAD Fixable Aqua Dead Cell Stain Kit (Thermo Fisher Scientific, 1:200) incubated with conjugated antibodies. Samples were acquired using an MA900 (Sony). For cell sorting, cells were stained using the same protocol and then sorted in 96 plates using the ultrapurity mode on an MA900 cell sorter (SONY). Data were analyzed with FlowJo or Kaluza software. Detailed gating strategies for individual markers are depicted in Fig. S2.

### Single-cell IgH sequencing

Sequences from RBD-specific MBCs from healthy donors were in part obtained from our previous analysis of RBD-specific MBCs after mRNA vaccination (Sokal et al., 2021a). All other sequences were obtained through single-cell sequencing. RBD-specific MBCs were single-cell sorted in 4 µl lysis buffer containing PBS (Gibco), dithiothreitol (Thermo Fisher Scientific), and RNAsin (Promega). A reverse transcription step was then performed using the SuperScript IV enzyme (Thermo Fisher Scientific) in 14 μl final volume (42°C 10 min, 25°C 10 min, 50°C 60 min, 94°C 5 min) with 4 µl of RNA in lysis buffer and random hexamers (Thermo Fisher Scientific). A PCR was further performed based on the protocol established by Tiller et al. (2008). Briefly, 3.5 μl of cDNA was used as template and amplified in a total volume of 40 μl with a mix of forward L-VH primer (Table S1 B) and reverse Cγ primer by using the HotStar Taq DNA polymerase (Qiagen) and 50 cycles of PCR (94°C 30 s, 58°C 30 s, 72°C 60 s). Then a second 50-cycle PCR using 5′AgeI VH primer mix and CHG-D1 3′ primer was performed before sequencing (Table S1 D).

PCR products were sequenced with the reverse primer CHG-D1 and read on ABI PRISM 3130XL genetic analyzer (Applied Biosystems). Sequence quality was verified with the CodonCode Aligner software (CodonCode Corporation).

### Computational analyses of VDJ sequences

Processed FASTA sequences from cultured single-cell V_H_ sequencing were annotated using Igblast v1.16.0 against the human IMGT reference database. Clonal cluster assignment (DefineClones.py) and germline reconstruction (CreateGermlines.py) were performed using the Immcantation/Change-O toolkit (Gupta et al., 2015) on all heavy chain V sequences. Sequences that had the same V-gene, same J-gene, including ambiguous assignments, and same CDR3 length with maximal length-normalized nucleotide hamming distance of 0.15 were considered as potentially belonging to the same clonal group. Mutation frequencies in V genes were then calculated using the calcObservedMutations() function from the Immcantation/SHazaM v1.0.2 R package. VH repartitions were calculated using the countGenes() function from the Immcantation/alakazam v1.1.0 R package. Further clonal analyses on all productively rearranged sequences were implemented in R. Graphics were obtained using the ggplot2 v3.3.3 and circlize v0.4.12 packages. Single-cell culture VDJ sequencing data are included in Table S1 C and have been deposited as Targeted Locus Study projects at DDBJ/EMBL/GenBank available as of the date of publication under the accession numbers KFVZ00000000–KFWQ00000000 (BioProject: PRJNA819082). The version described in this paper is the first version, KFVZ01000000–KFWQ01000000.

### Virus neutralization assay

Virus neutralization was evaluated by a focus reduction neutralization test. Vero E6 cells were seeded at 2 × 10^4^ cells/well in a 96-well plate 24 h before the assay. 200 focus-forming units of virus were preincubated with serial dilutions of heat-inactivated sera for 1 h at 37°C before infection of cells for 2 h. The virus/antibody mix was then removed and foci were left to develop in presence of 1.5% methylcellulose for 2 d. Cells were fixed with 4% formaldehyde and foci were revealed using a rabbit anti–SARS-CoV-2 N antibody (gift of Nicolas Escriou, Institut Pasteur, University Paris Cité, CNRS UMR 3569, Innovation Laboratory: Vaccines Unit, Paris, France) and anti-rabbit secondary HRP-conjugated secondary antibody. Foci were visualized by diaminobenzidine staining and counted using an Immunospot S6 Analyser (Cellular Technology Limited). Prepandemic serum (March 2012) was used as negative control for sera titration and was obtained from an anonymous donor through the ICAReB platform (BRIF code no. BB-0033-00062) of Institut Pasteur that collects and manages bioresources following International Organization for Standardization 9001 and NF S 96-900 quality standards.

The percentage of virus neutralization was calculated as (100−[(#foci sample/#foci control)*100]). Sera IC_50_ were calculated over seven fourfold serial dilutions from 1/10 to 1/40,000 using the equation log (inhibitor) vs. normalized response−−variable slope in Prism 9 (GraphPad software LLC).

### IFN neutralization assay

The blocking activity of anti–IFN-α2 and anti–IFN-ω auto-Abs was determined with a reporter luciferase activity. Briefly, HEK293T cells were transfected with a plasmid containing the firefly luciferase gene under the control of the human ISRE promoter in the pGL4.45 backbone and a plasmid constitutively expressing Renilla luciferase for normalization (pRL-SV40). Cells were transfected in the presence of the X-tremeGENE9 transfection reagent (Sigma-Aldrich, reference number 6365779001) for 24 h. Cells in DMEM (Thermo Fisher Scientific) supplemented with 2% FCS and 10% healthy control or patient serum/plasma (after inactivation at 56°C for 20 min) were either left unstimulated or were stimulated with IFN-α2 (reference number 130-108-984; Miltenyi Biotec) and IFN-ω (reference number SRP3061; Merck) at 10 ng/ml and 100 pg/ml or IFN-β (reference number 130-107-888; Miltenyi Biotech) at 10 ng/ml for 16 h at 37°C. Each sample was tested once for each cytokine and dose. Lastly, cells were lysed for 20 min at room temperature and luciferase levels were measured with the Dual-Luciferase Reporter 1,000 Assay System (reference number E1980; Promega) according to the manufacturer’s protocol. Luminescence intensity was measured with a VICTOR-X Multilabel Plate Reader (PerkinElmer Life Sciences). Firefly luciferase activity values were normalized against Renilla luciferase activity values. These values were then normalized against the median induction level for non-neutralizing samples and expressed as a percentage. Samples were considered neutralizing if luciferase induction, normalized against Renilla luciferase activity, was below 15% of the median values for controls tested the same day.

### Quantification and statistical analysis

Kruskal–Wallis test, Mann–Whitney test, and Wilcoxon test were used to compare continuous variables as appropriate (indicated in figures). Dunn’s correction was used for all multiple comparisons. Nonlinear regression was performed using semilog regression and Michaelis Menten model. A P value ≤0.05 was considered statistically significant. Statistical analyses were all performed using GraphPad Prism 9.0.

### Additional resources

ClinicalTrials.gov identifier: MEMO-CoV2, NCT04402892.

### Online supplemental material

Fig. S1 shows mRNA vaccines induce robust humoral response in patients with type I IFN deficiency, related to Fig. 1. Fig. S2 shows circulating MBCs are detected up to 6 mo after mRNA vaccination of patients with type I IFN deficiency, related to Fig. 2. Fig. S3 shows RBD-specific MBCs are GC derived in patients with type I IFN deficiency, related to Fig. 3. Table S1 provides more information about clinical data of the patients and values of anti-RBD IgG and IgA titers, sera IC_50_, flow cytometry data, and sequences of the IgV_H_. Table S1 also provides data about reagents/software used.

## Supplementary Material

Table S1 provides more information about clinical data of the patients and values of anti-RBD IgG and IgA titers, sera IC_50_, flow cytometry data, and sequences of the IgV_H_.
